# Peritoneal signalling improves hippocampal BDNF expression in aged mice

**DOI:** 10.18632/aging.204919

**Published:** 2023-07-19

**Authors:** Yoshinori Takei, Yoko Amagase, Atsushi Sugiyama

**Affiliations:** 1 Department of Pharmacology, Faculty of Medicine, Toho University, Japan; 2 Faculty of Pharmacy, Osaka Medical and Pharmaceutical University, Japan

**Keywords:** BDNF, vagus nerve, chemokine, rejuvenation, ageing

Ageing is associated with declining ability to learn new tasks. Brain-derived neurotrophic factor (BDNF) is a neurotrophin playing vital roles in the regulation of synaptic transmission and activity-dependent plasticity in the adult brain. Moreover, BDNF is essential for supporting adult hippocampal neurogenesis. These effects of BDNF are required for formation and maintenance of memory in the mature brain. The expression of *Bdnf* gene starts from early stage of development and is maintained through adult life. However, both expression of BDNF and its specific receptor TrkB decrease in the brain of the older adults [1]. Deficiency of hippocampal BDNF is observed in the older adults with diminished cognitive function. Decreased BDNF signalling in the brain of the older adults is thought to contribute to the age-dependent declining ability to learn new tasks.

Exercise improves cognitive function, which is most obvious in the older adults [2]. Moreover, exercise increases BDNF expression in various regions of the brain, most strongly in the hippocampus. Inhibition of BDNF signalling attenuates the effects of exercise on cognition. Thus, increased BDNF expression is necessary for exercise to improve cognitive function. However, it is still unknown how exercise induces BDNF expression in the hippocampus.

CX3CL1 (C-X3-C motif chemokine ligand 1) is a chemokine of which expression is increased with exercise [3]. A secretome analysis indicated that acute exercise altered expression levels of 938 genes in the muscle. In those, the number of genes encoding putative secreted proteins were 29. The *CX3CL1* gene was included in the 29 genes, and ELISA assay demonstrated increased blood levels of CX3CL1 after exercise. Moreover, CX3CL1 induces production of the crucial antioxidant enzyme heme-oxygenase-1 in macrophages expressing the CX3CL1 receptor CX3CR1 [4]. Free radicals, such as superoxide and hydroxyl radical, modify DNA, proteins and lipids, which induce cell senescence and accelerate ageing process. Reciprocally, antioxidants inhibit formation and propagation of free radicals, which in turn contributes to inhibiting or slowing the progression of ageing [5]. Therefore, CX3CL1-induced antioxidants might imply involvement of CX3CL1 in the anti-ageing mechanism activated by exercise. Contrary, serum CX3CL1 levels are also known to be increased in patients with type-II diabetes, obesity or rheumatoid arthritis. Therefore, it could be an interesting question to ask whether CX3CL1 in peripheral tissues can affect the cognition in aged mice, or not.

To identify effects of CX3CL1 in peripheral tissues on cognitive function, a soluble form of CX3CL1 including its chemokine domain was injected into the peritoneal cavity of aged mice [6]. Intraperitoneal administration of CX3CL1 in aged mice improved recognition memory declined with advancing age. In aged mice, peritoneal cells showed phenotypic changes, including increased population of cells expressing the senescence marker protein p16^*INK4a*^ and decreased phagocytic activity. Intraperitoneal administration of CX3CL1 recovered the age-associated changes in peritoneal cells, at least partially. Transplantation of peritoneal cells from CX3CL1-treated aged mice into the peritoneal cavity showed improved novel object recognition memory in aged mice. This suggests that peritoneal cells mediate the signalling from peritoneal CX3CL1 to improve recognition memory in aged mice.

Moreover, intraperitoneal CX3CL1 administration augmented the level of hippocampal BDNF expression and the number of hippocampal Type-2 neural stem cells (NSCs). Since Type-2 NSCs are intermediate cells from radial glial cell-like NSCs to mature neurons in the hippocampus [7], these results suggest that peritoneal CX3CL1 increases adult hippocampal neurogenesis. It is consistent with the essential role of BDNF in the adult hippocampal neurogenesis. The CX3CL1-induced BDNF expression was attenuated by vagotomy, a surgical division of the vagus nerve. This indicates that intact vagus nerve is essential for the signalling from peritoneal CX3CL1 to the hippocampus. Taken together with the involvement of peritoneal cells, this study showed that recognition memory in the older adults can be recovered by a unique connection among peritoneal cells, the vagus nerve and the hippocampus.

The vagus nerve is the longest nerve in the autonomic nervous system and conveys information to and from the viscera and brain. Recent studies have demonstrated that the vagus nerve is one of the major pathways by which signalling from peripheral tissues affects BDNF levels in the brain [8]. In our study, vagotomy had no effect on the basal level of hippocampal BDNF expression in aged mice [6]. However, the vagus nerve in aged mice still had a potential to augment BDNF expression in the hippocampus. These findings potentially suggest that age-associated alteration in vagus tone contributes to the diminished hippocampal BDNF expression in aged mice, thereby controlling progression of cognitive ageing. Further study should demonstrate the whole mechanism of cognitive ageing and provide effective methods for preventing the ageing.
